# Health‐Related Quality of Life and Everyday Functioning in the Flood‐Affected Population in Germany ‐ A Case Study of the 2021 Floods in West Germany

**DOI:** 10.1029/2024GH001135

**Published:** 2025-05-29

**Authors:** Nivedita Sairam, Anna Buch, Marie‐Luise Zenker, Lisa Dillenardt, Michaela Coenen, Annegret H. Thieken, Caroline Jung‐Sievers

**Affiliations:** ^1^ Section 4.4 Hydrology GFZ German Research Centre for Geosciences Potsdam Germany; ^2^ Institute of Geography University of Heidelberg Heidelberg Germany; ^3^ Institute of Environmental Science and Geography University of Potsdam Potsdam Germany; ^4^ Chair of Public Health and Health Services Research, Institute for Medical Information Processing, Biometry, and Epidemiology (IBE), Faculty of Medicine, LMU Munich Munich Germany; ^5^ Pettenkofer School of Public Health Munich Germany

**Keywords:** flash flood, disaster, recovery, psychological burden, flood impacts

## Abstract

Floods lead to adverse impacts not only in financial terms but also on the health of the exposed population. We report on health‐related Quality of Life (QoL) and functioning in the population affected by the 2021 flooding in Germany using an empirical survey data set. Health‐related QoL and functioning are represented by two scores–(a) The EuroQoL 5D Visual Analog Scale (EQ‐5D VAS) and (b) The 12‐Item World Health Organization Disability Assessment Schedule (WHODAS 2.0), respectively. By applying an incremental linear regression model and Machine Learning models, we infer that health‐related QoL and functioning are strongly negatively related to the psychological burden from those being affected by the flooding. This includes how often they think about the traumatic event. Home owners were found to have worse QoL and functioning than tenants. Household income and the status of repair/reconstruction of flood damages—in specific, insurance benefits, private donation and satisfactory claims compensation are associated with high health‐related QoL and functioning. These findings highlight the importance of strengthening the health‐related QoL of flood affected populations and emphasizes the strong association between recovery and health‐related QoL and functioning of flood‐affected populations.

## Key Points

- Incremental linear regression and Machine Learning models are applied on an empirical data set from a population affected by flooding in July 2021
- Psychological burden of people from being affected by flooding is strongly associated with decline in health‐related Quality of Life (QoL) and functioning
- Household income and the status of repair of flood damages are associated with health‐related QoL and functioning in flood‐affected people

## 1 Introduction

Globally, floods are the leading climate‐related threat to people in terms of occurrences, financial damage and number of people affected: between 2000 and 2019, floods were the most common disaster worldwide affecting 1.65 billion people (van Loenhout & McClean, 2020). Changing climate and growing cities contribute to rising flood risk (Rentschler et al., 2022). In addition to the strong research and practice focus on flood impacts to built‐up assets, floods exert profound effects on affected populations, manifesting in both direct and indirect consequences.

The most conspicuous intangible consequence of flooding is its impact on human health (Du et al., 2010). It can lead to more obvious physical health problems such as injuries or infections, and can also have a negative impact on a person's mental health in the short and long run (Ahern et al., 2005; Alderman et al., 2012). In specific, depression, anxiety and other mental health disorders can impair a person's ability to carry out daily activities and reduce their perception of well‐being (Serrano‐Aguilar et al., 2009). These consequences lead to a decrease in health‐related Quality of Life (QoL) and functioning of the flood‐affected populations. The World Health Organization (WHO) defines QoL as the individuals' perception of their position in life in the context of the culture and value systems in which they live and in relation to their goals, expectations, standards, and concerns. In our study, we use the standardised instruments, EQ‐5D VAS (EuroQol 5D Visual Analog Scale) to measure the health‐related QoL and WHODAS (WHO Disability Assessment Schedule) 2.0 to measure the functioning of flood‐affected individuals. Health‐related QoL is defined as the impact of the health aspects of an individual's life on that person's QoL, or overall well‐being. It is also used to refer to the value of a health state to an individual (Brazier et al., 2007; Shah, 2017). Functioning is defined as an objective performance in a given life domain and WHODAS 2.0 is a standardised way to measure each function of an individual—at body, person or society level (Üstün, 2010). Psychological burden due to flooding captures to what extent the respondents feel burdened by the flood. It is a self‐reported aspect of recovery referring to the degree the respondents have returned to their original (pre‐flood) state (Bubeck & Thieken, 2018).

Various studies have been conducted to examine factors that may influence the health burden of people affected by flooding. Flood‐related health outcomes have been found to be associated with the flood hazard characteristics (Babcicky & Seebauer, 2021; Bubeck & Thieken, 2018; Ramesh et al., 2023). Certain socio‐demographic factors have often been identified to affect the health burden of flooding, for example, gender (Mason et al., 2010; Norris et al., 2002; Paranjothy et al., 2011; Tunstall et al., 2006) or income (Graham et al., 2019; Lamond, 2014; Mason et al., 2010; Paranjothy et al., 2011; Tunstall et al., 2006). Very few studies on flood‐related health outcomes account for confounding controls and quantify the role of post‐disaster relief and response (Apel & Coenen, 2020; Bubeck & Thieken, 2018). These studies implemented regression approaches with logit and probit model definitions.

Flood impacts, both socioeconomic and health related, often create cascading effects that worsen the overall consequences, for example, the cascading effects of the immediate and short‐term flood impacts such as injuries on long‐term physical health impacts such as musculoskeletal and cardiovascular diseases; the impact of financial losses on mental health consequences such as depression and Post‐Traumatic Stress Disorder (PTSD) leading to an overall reduction in health‐related QoL (Babcicky & Seebauer, 2021; Berry et al., 2018; Zenker et al., 2024). Flood risk response such as evacuation was found to pose a serious threat to the mental health of the flood‐affected population (Armstrong et al., 2017; Wind et al., 2013). Flood experience was found to influence awareness and preparedness (Barendrecht et al., 2019), which acts also a potential flood disaster mental health intervention (Hudson, 2023). Mental health and health‐related QoL of the flood‐affected population was also found to be strongly related to the efficiency of claim compensation by insurance (Carroll et al., 2010; Mulchandani et al., 2019; Tunstall et al., 2006) and social and emotional support received by the flood‐affected population (Guilaran et al., 2018; Park et al., 2021).

Due to unavailability of data at the micro‐scale (individuals), the challenge to attribute these impacts to potential drivers (e.g., interconnected social processes and preparedness) still remains. Owing to the large number of potential drivers of flood impacts and their interactions, Machine Learning based models have proven to be useful in the context of modeling economic flood impacts (Schoppa et al., 2020; Schröter et al., 2014). To the best of our knowledge, such methods have not been implemented in the context of flood‐related health impacts. Hence, our study applies machine‐learning methods next to traditional statistical approaches.

Our study contributes to overcoming the lack of empirical evidence that explains the health‐related QoL and functioning of flood‐affected populations. We apply an incremental linear regression model and Machine Learning (ML) models on empirical household survey data from the 2021 flood event in Germany, which has been the most damaging flood in Germany for decades. Based on the data‐driven models, we elucidate associations between health‐related QoL and functioning of the flood‐affected population and their socioeconomic attributes, experiences during the flood event, flood consequences and recovery.

## 2 Data and Methods

### 2.1 2021 Floods in Germany

Regions in Western Germany, in specific Rhineland‐Palatinate and North Rhine‐Westphalia received heavy rainfall in July 2021 due to the low‐pressure system “Bernd” leading to severe flooding that affected more than 85 thousand people. Parts of Rhineland‐Palatinate received a record rainfall of 100 mm in 72 hr and parts of North Rhine‐Westphalia received more than 175 mm rainfall in 48 hr (Kron et al., 2022; Tradowsky et al., 2023) leading to fast‐rising water levels and flash flooding in some catchments. In particular, the catchments of the river Ahr (ca. 900 km^2^) in Rhineland‐Palatinate and Erft (ca. 1800 km^2^) and Rur (more than 2000 km^2^) in North Rhine‐Westphalia were most affected; all originate in the Eifel middle hills. The total number of affected buildings was estimated to be more than 9000 along only the Ahr Valley (Birkmann et al., 2023). The event injured 823 people (Schäfer et al., 2021), and killed 186 people (Thieken et al., 2023). The actual damage sum is estimated to be around 33 billion euros in Germany alone (BMI, 2021).

### 2.2 Survey Population

**Figure 1:**
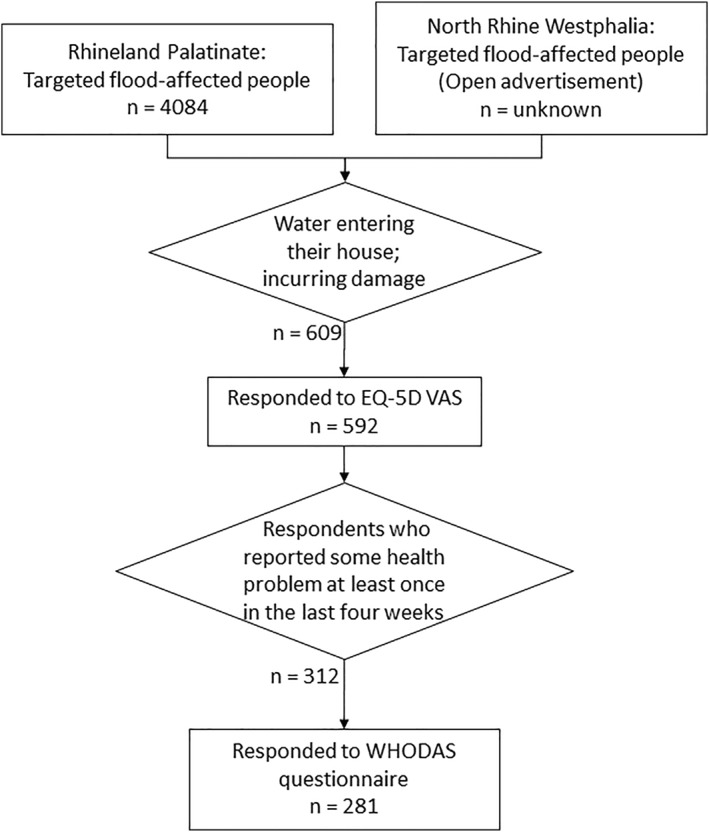
Flow Chart of the target respondents reported according to the STROBE statement.

In Rhineland‐Palatinate, the respondents were recruited based on a list of households from the county of Ahrweiler that applied for emergency relief; every third household was invited to answer the survey. In North Rhine‐Westphalia, the survey was advertised through social media. The survey was undertaken in November and December 2022, that is, 16–17 months after the flood event. It should be noted that the survey was conducted during the Corona pandemic. Only households that experienced the flood event with water entering the house resulting in financial damage were of interest for the survey. Hence, the survey did not have a control group of people who did not experience flooding. Only respondents over the age of 18 were allowed to partake in the survey. It took around 40–50 min to complete the questionnaire. The surveys are anonymous where the respondents were only requested for an optional 3‐digit postcode. Participants were invited to take part in the survey only if they felt sufficiently resilient, with a warning about the potential risk of re‐traumatization. This is important because answering the questionnaire can trigger distressing memories. In addition, the respondents were informed in detail before the survey about the motivation behind the collection of the data, what the data would be used for and that, under certain circumstances, decisions could be made on the basis of the participants' answers, for example, at a political level, which could be disadvantageous for the participants. Since the survey was conducted online, immediate professional help was not available. To address this, we included warning messages and provided contact information for the BDP (Professional Association of German Psychologists) as well as local support resources before, during (especially, before and after the questions concerning flood impacts, and after the survey (see, Table S1 in Supporting Information S1). Additionally, since the subjects were highly personal and sensitive, the topics and aims of the survey were discussed with the ethics committee of the University of Potsdam. The survey was offered in German language which might introduce a selection bias since potential vulnerable groups may have difficulties answering questionnaires in German language and might be left out of the sample. We obtained a voluntary sample of 277 respondents from Rhineland‐Palatinate and 332 from North Rhine‐Westphalia. The response rate in North Rhine‐Westphalia is difficult to estimate as we do not know how many people noticed the advertisement. In Rhineland Palatinate, 4084 invitation letters were successfully delivered, resulting in a response rate of approximately 7% (see, Figure 1).

### 2.3 Material ‐ Survey Questionnaire

The online survey consisted of 109 questions (see, Table S1 in Supporting Information S1) covering the following topics:

1. Background information on the respondents:
  (a) Socioeconomic characteristics—age, gender, income, education, members in household–children, elderly members and home ownership.
  (b) Building Characteristics–building type, building quality, footprint size

The socioeconomic classification based on Plapp (2003) was applied considering the living condition (ownership, building type), living space (footprint size of the house) and the highest education level of the household.

2 Flood impact‐related aspects:
  (a) Awareness and preparedness—past flood experience, warning characteristics, implementation of emergency and property‐level flood risk adaptation measures as well as flood insurance;
  (b) Flood event characteristics and response—inundation depth and duration, flow velocity (intensity), contamination;
  (c) Impacts–evacuation, damage to building and contents, injury to self, injury to friends/family and death of friends/family;
3 Recovery from Floods—source and form of financial and social support, status of repairing the house and repurchasing the contents, psychological burden from the flooding (the psychological burden measures subjective perception and is not on a validated scale).
4 Health status of the flood affected population:
  (a) Self‐rated health from the EuroQol 5D Visual Analog Scale (EQ‐5D VAS—[0,100]):
    EQ‐5D VAS requires minimal cognitive effort from the respondent—they are asked to rate their health on a visual scale of 0–100, with 0 being the worst health to 100 being the best health. It is straight‐forward to administer EQ‐5D VAS as a part of surveys such as in our case. EQ‐5D VAS is self‐rated, meaning, it provides the perception of the respondent regarding their health status.
  (b) The sum score from the 12‐item questionnaire of WHODAS (WHO Disability Assessment Schedule) 2.0 to assess functioning.

WHODAS 2.0 assesses functioning and disability of the respondents irrespective of health conditions. The WHODAS 2.0 consists of 12 items to measure the impact of a health condition or traumatic experience on an individual's ability to perform day‐to‐day activities in six domains: cognition, mobility, self‐care, getting along with others, life activities, and participation in society (Andrews et al., 2009). The level of difficulty is measured in a scale of 1–5 (None, Low, Moderate, Extreme, Very Extreme/Not Possible).

The WHODAS 2.0 was only targeted toward flood‐affected people who reported that they had some health problem (mental, physical illness, emotional or drug‐related) at least once in the last 4 weeks.

Out of the 609 completed surveys, 592 respondents furnished information on EQ‐5D VAS; out of the 312 target respondents, 281 furnished information on WHODAS 2.0 (see, Figure 1). Respondents who have not answered health‐related questions were removed from the respective analysis. The missing values in other covariates were imputed using the predictive mean matching method.

### 2.4 Analysis of Covariates

In order to identify the covariates associated with health‐related QoL of flood‐affected populations, simple linear regression models and ensemble of ML models are built on the empirical survey data set described in Section 2.2. In these models, the standardised covariates derived from the survey responses are regressed against the health‐related QoL and functioning represented by EQ‐5D VAS and WHODAS scores.

#### 2.4.1 Incremental Linear Regression Model Specification

In our study, variables representing health‐related QoL of the flood‐affected population, that is, EQ‐5D VAS and WHODAS score comprises the dependent variable. In order to identify the association of the socioeconomic attributes of the flood‐affected population, their flood experience and flood recovery, three linear models with incremental complexity are conceptualized.

1. Basic Model: Health‐related QoL is regressed against residential building attributes and socioeconomic characteristics of the respondents.
2. Extended Model (+Flood): Health‐related QoL is regressed against residential building attributes, socioeconomic characteristics and also the flood experience of the respondent comprising the event characteristics, preparedness and impacts.
3. Comprehensive Model (+Flood Recovery): Health‐related QoL is regressed against residential building attributes, socioeconomic characteristics and also the flood experience of the respondent comprising the event characteristics, preparedness, impact and recovery attributes.

The covariates in the three models are chosen based on a forward selection and backward elimination method based on the Akaike Information Criterion (AIC).

#### 2.4.2 Machine Learning (ML) Models to Identify Factors Associated With Health‐Related QoL

In order to model linear and non‐linear interaction effects between potential risk factors, also called features (independent variables), and the health‐related QoL and functioning in flood‐affected populations, we apply three ML models–Elastic Net, Random Forest and XGBoost (*scikit.learn* python package, Pedregosa et al., 2011).

Elastic Net (Zou & Hastie, 2005) is a linear regression model that combines the L1 (Lasso) and L2 (Ridge) regularization. This helps in feature selection while handling correlated features and also reduces overfitting by allowing flexibility to tune the hyper‐parameters favoring either Ridge or Lasso regularizations. Random Forest (Breiman, 2001) is a tree‐based model which itself is an ensemble (decision trees) learning method. In the learning process, the data points are randomly split into subsets based on bootstrapping with replacement and each tree is built on the subset across a randomly selected subset of variables. Averaging the prediction across all the constructed trees eliminates overfitting. eXtreme Gradient Boosting (Chen & Guestrin, 2016) is also an ensemble of decision trees. In contrast to Random Forest, XGBoost uses a gradient boosting algorithm where each new tree is built by correcting the errors from the previous trees. XGBoost penalizes overfitting using both L1 and L2 regularizations.

The three models are preferred over other ML algorithms due their handling of correlated features (Elastic Net), robustness (Random Forest) and good performance despite missing feature values (XGBoost). For model training we cross‐validate each model by using 10‐folds and repeating the cross‐validation procedure 5 times in order to find for each model its best set of hyperparameters. The k‐fold repeated cross‐validation reduces the tendency of the models to overfit and provides model performance metrics. After model training we apply Permutation Feature Importance to identify the factors that are associated with the health status of the flood‐affected population. We visualize the feature importances of all three models separately in order to show potential similarities and differences in the predictive importance of each factor across the models.

Permutation Feature importance measures the model's performance by repeatedly shuffling the values of one predictor at a time. The model setup (i.e., hyper‐parameters) with the least Mean Absolute Error (MAE) is used to derive feature importance based on nested cross‐validation (train and test data points are independent of each‐other). The importance of each predictor is the average reduction in model performance (i.e., MAE) when the predictor is shuffled (averaged over 10‐folds). Since permutation feature importance is model‐agnostic, it helps evaluate the significance of the individual predictors in an multi‐model ensemble. Partial Dependence Plots (PDPs) provide the marginal effect of a feature on the predicted outcome while keeping other features constant.

## 3 Results

### 3.1 Characteristics of the Flood‐Affected Population

#### 3.1.1 Socioeconomic and Building Characteristics

The sample of respondents had 42% male and 58% female respondents with 72% of the respondents earning more than 2600 euros per month as the net household income (comparative with average German household salary) (Destatis—Statistisches Bundesamt, 2020). The majority of the households were 2‐person households with 24% of the respondents above 65 years of age. The majority of the respondents were owners of the building having an average building footprint size of 153 square meters. These attributes led to a fairly high average socioeconomic status of the flood‐affected population (Plapp, 2003). However, the quality of the buildings before the floods were reported to be mostly poor and needing renovation.

#### 3.1.2 Flood Event ‐ Preparedness and Impact

Almost all the households that were impacted by the 2021 flood event had no previous flood experience (74.1%). The warning source indicator derived based on the reliability of the warning source and the warning lead time has a small mean value–44% households did not receive a warning. Majority of the households implemented simple emergency measures such as protecting documents and valuables. Around 50% of the households implemented the adaptive measure of purchasing flood insurance before the event.

The flash flood event resulted in fast rising water depths leading to severe damages to the exposed buildings and population. For instance, the velocity of the flood was perceived to be torrential with an average depth of 1.3 m 50% of the flood‐affected respondents reported that an average man would have been swept away by the flood. Approximately 63% of the survey respondents were evacuated at some point during the flood event. Twenty‐seven percent were evacuated before or during the onset of the event and 36% were evacuated after the flood event.

The households reported an average damage of 253,000 euros to buildings and 51,000 euros to household contents. Ten percent of the respondents suffered from injuries to themselves, 25% experienced injury among their families and friends and 11% suffered from death of family or friends due to the flood event. Additionally, the majority (65%) were worried about the wellbeing of their family and friends because of the flood event.

#### 3.1.3 Recovery From Flooding

Several aspects concerning recovery from the flooding are considered. Repurchasing/repairing damaged household contents and repairing the building was quantified on a Likert scale (1–6). The responses pointed out that at the time of the survey (16–17 months after the event), approximately 50% of the houses still have to make significant repairs to buildings and repurchase damaged contents. This aligns with the metric that claims from just 60% of the respondents were completed at the time of the survey. The level of satisfaction with the claims settlement was also centered at around 50%. Among the other forms of financial help, a significant share of respondents (71% and 54%) received immediate aid and insurance benefits, respectively. 77% and 59% of the respondents received support from family/friends and the community respectively. However a smaller share of 25% of the respondents received support from the first responders and welfare organizations.

#### 3.1.4 Self‐Reported Health of Flood‐Affected Population

The flood‐affected population from the 2021 event exhibited a mean(sd) EQ‐5D VAS score of 67.73(1.03) and a mean(sd) WHODAS 2.0 score of 26.98(10.39). Considering the spread of WHODAS 2.0 responses between the 25th and 75th percentile, the flood‐affected individuals with health problems exhibit none to low difficulty in the domain of self‐care (Q8, Q9); none to moderate difficulty in the domains of cognition (Q3, Q6), mobility (Q1, Q7), life activities (Q2, Q12) and getting along with others (Q10, Q11) and low to extreme difficulty in participation in society (Q4, Q5) (See Figure S1 in Supporting Information S1). In general, the level of difficulty experienced by flood‐affected individuals across all domains of functioning are consistent (Cronbach's alpha–0.924).

### 3.2 Factors Associated With Health‐Related QoL in Flood‐Affected Populations

#### 3.2.1 Factors Identified Based on Incremental Linear Regression Model

The incremental specification of the linear regression model resulted in three sets of covariates (chosen by forward‐selection and backward elimination based on AIC) that are associated with the health‐related QoL in flood‐affected populations.

**Figure 2:**
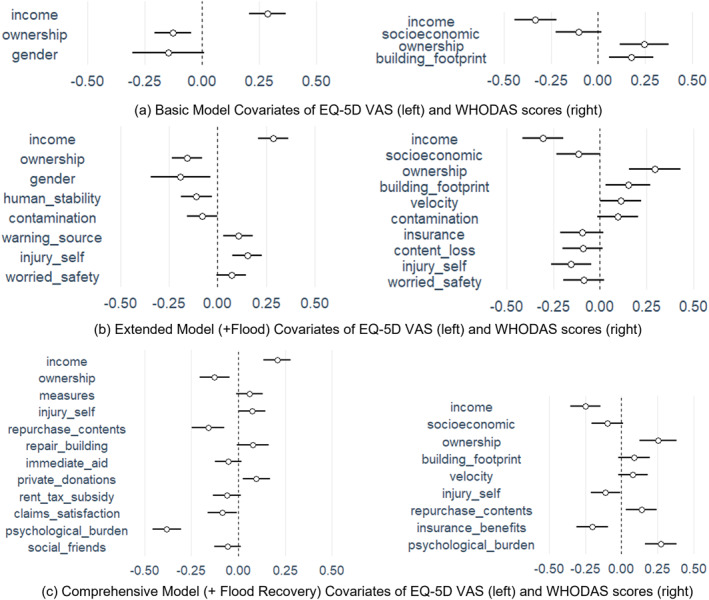
Coefficients of the Basic (a), Extended (b) and Comprehensive (c) Linear Regression Model with targets EQ‐5D VAS and WHODAS score (the range represents 0.95 confidence interval of the coefficients).

The basic model specification considers only the socioeconomic attributes and residential building characteristics of the flood‐affected populations. Both EQ‐5D VAS and WHODAS scores are associated with the household income and ownership (Figures 2a–2c). Additionally the EQ‐5D VAS score varies by the gender of the responder and the WHODAS score is associated with the household's socioeconomic status and building footprint size (Figures 2a–2c). The Extended Model specification includes flood‐related attributes (event characteristics, preparedness and impact attributes) (see, Figure 2b). We find that the socioeconomic attributes are retained. Additionally, the reliability of the warning (source of warning) is associated with the EQ‐5D VAS score and the presence of flood insurance is associated with the WHODAS score. Household content damages are associated with only the WHODAS score. Whereas, flood intensity indicators, such as velocity/human stability and contamination, and flood impact aspects, such as injury and anxiety regarding safety of friends and family are associated with both the EQ‐5D VAS and the WHODAS scores.

Finally, we include the flood‐recovery related aspects leading to the comprehensive model specification (see, Figure 2c). Income is positively (negatively) associated with EQ‐5D VAS (WHODAS) score, whereas home ownership was found to have a negative (positive) association with the EQ‐5D VAS (WHODAS) score. Respondents who were injured during the flood event were found to exhibit a lower (higher) EQ‐5D VAS (WHODAS) score. Recovery‐related aspects including status of repurchase of damaged household contents, financial help in the form of insurance payouts and private donations and satisfactory insurance claims settlement were found to be positively (negatively) associated with EQ‐5D VAS (WHODAS) score of the flood‐affected populations. Receiving social support from friends, family and neighbors was found to be negatively associated with EQ‐5D VAS. However, the other sources of social support were not associated with either EQ‐5D VAS or WHODAS.

Additionally, the data shows a few weak associations–there is weak evidence for variability in EQ‐5D VAS scores across genders (Figures 2a–2c). The implementation of emergency measures, availability of immediate aid and rent/tax subsidies and completion of building repairs were found to be weakly related to the EQ‐5D VAS scores (Figures 2b and 2c). The variability in WHODAS scores is only weakly explained by socio‐economic status of the household, size of the building footprint and also the velocity of the experienced flood (Figures 2a and 2b).

#### 3.2.2 Feature Importance From ML Models

**Figure 3:**
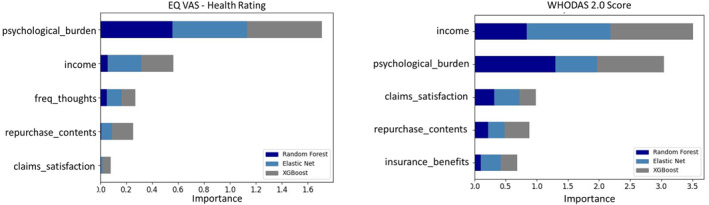
Top 5 Features associated with EQ‐5D VAS and WHODAS scores–Importance scores are the reduction in model performance in the absence of the feature.

**Figure 4:**
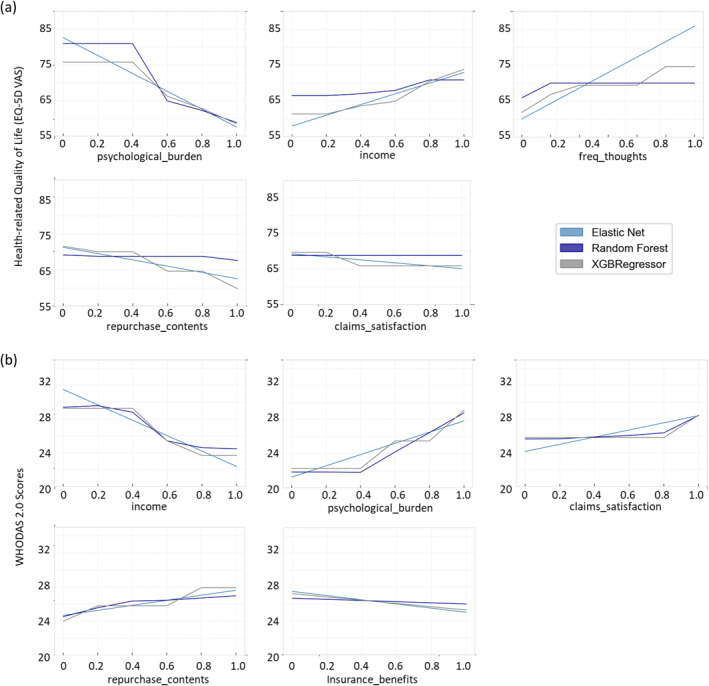
Partial Dependence Plots showing the association of the top 5 features with health‐related Quality of Life and functioning. The standardised value of the features (i.e. between 0 and 1) are shown on the *x*‐axis and the EQ‐5D VAS (a) WHODAS (b) scores are shown on the *y*‐axis.

By applying the three ML models and permutation feature importance (see Section 2.3.2), we derive the top 5 features that are associated with the health‐related QoL and functioning–EQ‐5D VAS and WHODAS scores (Figure 3). The relationship between the individual features and the EQ‐5D VAS and WHODAS scores are presented using the PDPs (Figure 4).

The relationship between health‐related QoL and functioning and the features are elucidated by the partial dependence plots (PDPs; Figure 4). In agreement with the results from the linear regression model, the ML models also recognized income to be positively (negatively) associated with EQ‐5D VAS (WHODAS) scores; psychological burden from the flood event was found to be negatively (positively) associated with EQ‐5D VAS (WHODAS) scores. Individuals with satisfactory claims settlement and higher state of repurchasing damaged household contents were found to have higher (lower) EQ‐5D VAS (WHODAS) scores. Receiving insurance benefits has a positive impact on the EQ‐5D VAS scores. Complementing the results from linear regression, the ML‐models revealed that individuals who thought about the flood event more frequently exhibited lower EQ‐5D VAS scores, meaning, lower perception of their health‐related QoL.

The comprehensive linear regression model and the three ML‐based models were validated based on a 10‐fold cross‐validation. All the models performed with comparable error metrics. For EQ‐5D VAS prediction (range of 0–100), the Mean Absolute Errors (MAE) ranged between 16 and 18 and Root Mean Square Error (RMSE) between 21 and 22.5. In the case of WHODAS prediction (range of 12–60), the Mean Absolute Errors (MAE) ranged between 7 and 7.8 and RMSE between 9 and 9.6.

## 4 Discussion

The study uses a survey data set *n* = 592 (EQ‐5D VAS) and *n* = 281 (WHODAS 2.0) from populations affected by the 2021 floods in Rhineland‐Palatinate and North Rhine‐Westphalia, Germany. Association between health‐related QoL and functioning of the flood‐affected population and their socioeconomic attributes, experiences during the flood event, flood consequences and recovery are derived using an incremental linear regression and ML models.

The health‐related QoL measured by the EQ‐5D VAS score (mean: 67.73; SE: 1.03) of the flood‐affected population is lower in our sample in comparison to the EQ‐5D VAS score of the general population in Germany (mean: 75.3, SE: 0.2) (Szende et al., 2014). The difference seems to be of similar magnitude across all age groups and genders of the flood‐affected population (see, Figure S2 in Supporting Information S1). The sum of scores on the 12‐item WHODAS 2.0 obtained from the flood‐affected population (mean: 26.98, SD: 10.39) can be regarded comparable or even marginally higher than the score obtained from patients with stress disorder (mean: 21.1, SD: 6.3) and anxiety (mean: 24.7, SD: 8.5) (Axelsson et al., 2017). It should be noted that the WHODAS 2.0 questionnaire was only targeted at flood‐affected respondents who reported that they suffered from some physical or mental disorders at least once in the last 4 weeks (Figure 1).

The comprehensive linear regression model suggests that among socioeconomic factors, only income and home ownership are associated strongly with health‐related QoL of flood affected populations (Figure 2c). We also find that flood‐affected homeowners exhibit lower health‐related QoL in comparison to tenants (Figure 2c). A study conducted by Trüdinger et al. (2023) demonstrated that tenants are more likely to relocate after a flood. The findings could also be related to a sense of strong attachment to the place. This is especially relevant in the case of a damaging event such as the 2021 flooding where 50% of the exposed buildings suffered more than 159,000 euros of building damage (Table 1). Gender is a risk‐factor contributing to health‐related vulnerability in the context of disasters (Mason et al., 2010; Paranjothy et al., 2011; Zenker et al., 2024). However, in our study, gender was found significant only in the basic linear model, not considering flood‐related attributes. Individuals who have been injured during the flood event also exhibited low health‐related QoL (Figure 2c). 820 persons were reported as injured during the flood event (as of July 21. 2021) leading to significant impact on the health and functioning of the general population in the flood‐affected regions. Getting injured or sick during the flood event was also shown to be a risk factor for developing posttraumatic stress disorder (Zenker et al., 2024).

**Table 1:** Description of Respondents' Socioeconomic Attributes, Residential Building Characteristics, Flood‐Related Aspects and Health Indicators

Category	Explanation	Variable name	Range, mean, median
(I) Socioeconomic and Residential Building Characteristics	Gender	Gender	Male–42%
			Female–58%
	Age of the respondent (years)	Age	18:19%–0.16%
			20:29%–2%
			30:39%–11.3%
			40:49%–19.9%
			50:59%–29.3%
			60:64%–13.5%
			65:69%–7.6%
			70:74%–9.1%
			75:79%–3.7%
			≥80–3.37%
	Household Size (number of persons living in household)	Household_size	[1,12], 2.46, 2
	Household Monthly Income (euros)	Income	Under 900%–0.8%
			900:1299%–4.4%
			1300:1499%–3.2%
			1500:1999–7.1%
			2000:2599%–14.7%
			2600:3599%–25.5%
			3600:4999%–23.4%
			≥5000–20.9%
	Socioeconomic status–(computed based on ownership, footprint size, building type, education; see, Plapp, 2003, p. 3–very low; 13–very high)	Socioeconomic	[6, 13] 10.14, 10
	Ownership (ordinal)	Ownership	Tenant–25.4%
			Flat owner–8.3%
			Building owner–66.3%
	Building Type (nominal)	Building_type	Single family: 46%
			Multi‐family: 26%
			Semi‐detached: 28%
	Building Quality before the flood (1–very good; 6–very poor)	Building_quality	[1, 6] 4.63, 5
	Building footprint size (square meters)	Building_footprint	[30,1000] 153, 100
(II) Flood Risk Awareness/Preparedness	Flood Experience:	Flood_experience	[0, 5] 0.35, 0
	Number of floods experienced before the 2021 event. 0–no flood experience; 5–experienced more than 4 events.		
	Warning source: Indicator calculated based on the authenticity of the different sources of warning (0–no warning; 1–own observation; 2–via contacts; 3–media; 4–authority)	Warning_source	[0, 4] 1.35, 1
	Warning lead time (hours): Time between receiving the warning and onset of the flood event	Warning_time	[0, 48] 3.26, 0
	Measures: Implementation of one or more emergency measures (0–no; 1–yes).	Measures	[0,1] 0.75, 1
	List of measures: Documents and valuables secured; Furniture and movable objects raised or moved to a safe place; Oil tanks or containers with other hazardous substances secured; Water pumped out or skimmed off; Animals brought to safety; Vehicles driven onto flood‐proof terrain; Protect the building itself against water penetration, for example, by sealing doors, windows, drains and other openings; Divert water through measures on the site, for example, digging trenches, building sandbag walls, mobile walls, etc.; Get help from outside (fire brigade, friends, etc.); Electrical devices unplugged, secured or sockets taped off; Electr. installations, permanently installed building parts (doors, etc.) secured or dismantled; Gas/electricity switched off; Gas/electricity cut off centrally by municipal utilities.		
	Insurance status of households against heavy rain and floods (0–no; 1–yes)	Insurance	[0, 1] 0.5, 0.5
(III) Flood event Characteristics and Response	Maximum Water Depth (cm) (negative values indicate basement flooding)	Water_depth	[−229, 700] 131.85, 120
	Duration of Inundation (hours)	Duration	[1, 240] 55, 36
	Contamination Indicator–weighted average of contaminants in flood water. (0–no contamination; weight[oil/gasoline] = 3; weight[chemicals] = 2; weight[sewage] = 1)	Contamination	[0, 6] 2.37, 1
	Maximum Velocity (0: quietly flowing; 6–torrential)	Velocity	[0, 6] 3.92, 4
	Human Stability: Combination of velocity and water depth representing maximum flood intensity (1–a man could stand effortlessly; 2–have to strain to stand; 3–swept away 4–too deep to stand)	Human_stability	[1, 4] 2.57, 3
(IV) Impact	Evacuation: Whether the household was evacuated before/during the event (1–yes; 0–no)	Evacuation_b	[0, 1] 0.27, 0
	Evacuation: Whether the household was evacuated after the event (1‐ yes; 0–no)	Evacuation_a	[0, 1] 0.36, 0
	Damage to building (euros)	Building_loss	[400, 5000000] 253433, 159000
	Damage to contents (euros)	Content_loss	[30, 600000] 51147, 30000
	Injury to self (1–yes; 2–no)	Injury_self	[1, 2] 1.91, 2
	Injury to family or friends (1‐ yes; 2–no)	Injury_family	[1, 2] 1.75, 2
	Feeling anxious about safety of family or friends (1–yes; 2–no)	Worried_safety	[1, 2] 1.35, 1
	Death of family/friends (1–yes; 2–no)	Death_family	[1, 2] 1.89, 2
(V) Recovery	Repair/Re‐purchase all damaged contents (1–all damages contents were repaired or repurchased; 6–there are major repairs/purchases to be done)	Repurchase_contents	[1, 6] 3.19, 3
	Repair the building damages completely (1‐ the building is completely repaired; 6–there are still major repairs to be done)	Repair_building	[1, 6] 3.20, 3
	Thinking about flood (1–several times a day; 6–not once in the last 6 months)	Freq_thoughts	[1, 6] 2.70, 3
	Burden of flood (1–doesn't bother at all; 6–still weighs heavily)	Psychological_burden	[1, 6] 4.03, 4
	Claim settlement complete (1–yes; 0–no)	Claims_completion	[0, 1] 0.57, 1
	Level of satisfaction with the claim settlement (1–very satisfied; 6–very dissatisfied)	Claims_satisfaction	[1, 6] 3.18, 3
	*Form of financial help received (1*–*yes; 0*–*no):*		
	Immediate help	Immediate_aid	[0, 1] 0.71, 1
	Oil spill response assistance	Oilspill_response	[0, 1] 0, 1
	Hardship reconstruction assistance	Hardship_assistance	[0, 1] 0.11, 1
	Private donations	Private_donations	[0, 1] 0.30, 1
	Insurance benefits	Insurance_benefit	[0, 1] 0.54, 1
	Rent reduction; preferential treatment by energy suppliers, tax relief.	Rent_reduction	[0, 1] 0.07, 1
	*Form of social support (Donations in cash, kind or support staff) received (1*–*yes; 0*–*no):*		
	Support from family, friends and neighbours	Social_friends	[0,1] 0.77, 1
	Support from the community, volunteers or church	Social_community	[0,1], 0.59, 1
	Support from police, first responders and welfare organizations	Social_organization	[0,1], 0.25, 0
(VII) Health Status	Health status (EQ‐5D VAS; 0–100)	EQ‐5D VAS	[1,100] 68, 74
	WHODAS 2.0 [sum of the score from 12‐item questionnaire]	WHODAS 2.0	[12,55] 27, 25

The ML models help to capture nonlinear relationships between the features and health‐related QoL and functioning of the flood‐affected population (see, Figure 4). We see an almost linear increasing relationship between income and EQ‐5D VAS with a thresholding effect at 20% (XGBoost and random forest) suggesting that financially resilient individuals are associated with better health‐related QoL and functioning. This is in alignment with the difference in life expectancy of men in Germany between the highest and the lowest income groups (Nowossadeck et al., 2019).

The majority of the factors associated with the health‐related QoL of flood‐affected population belonged in the “Recovery” aspect of flood risk management (see, Table 1 and Figure 3). The largest factor negatively associated with the health‐related QoL is the perceived psychological burden of flooding and additionally, how rarely the individuals think about the traumatic event (Figure 4). It should be noted that psychological burden is measured as subjective perception and does not refer to a validated scale. The two non‐linear models (XGBoost and random forest) exhibit a thresholding effect of psychological burden at 40% (respondents experiencing some level of burden from the flood) and a steep decline in health‐related QoL and functioning beyond this level. This is supported by findings from Zenker et al. (2024), who found that 28% of the surveyed residents in the affected area showed indications of PTSD 1 year after the flood event based on a validated screening scale. This shows the general need to strengthen the health‐related QoL of flood‐affected populations and in specific, focus on reducing the psychological burden for individuals.

Recovery indicators, such as the status of repurchasing the damaged household contents and repairing the damaged parts of their building, were positively associated with health‐related QoL. We observe a uniform distribution of households across the several levels of repair/repurchase of damaged contents 1.5 years after the event (see, Figure S3 in Supporting Information S1). This indicates various levels of recovery across the flood affected households. Recovery of building and household contents is not only dependent on the socioeconomic characteristics of the household but also supported by risk transfer measures and donations. Successful risk transfer mechanisms such as receiving satisfactory claims compensation, insurance payouts and private donations were found to be positively associated with health‐related QoL (Figure 2c). The flood event is expected to result in a total of 8.2 billion euros of insured losses (Gallin, 2021). The biggest proportion of this (7.7 billion euros) will be toward residential building and content damages. At the time of the 2021 floods, only 46% of the German households had taken out an insurance against flood damages (GDV, 2024). However, owing to growing awareness and lobbying, more than two thirds of German households support the introduction of compulsory flood and natural hazard insurance (ZEW, 2022). Our results also highlight the impact of insurance and recovery mechanisms not only on improving financial resilience but also to strengthen the health‐related QoL of flood‐affected populations to reduce their psychological burden. Though we account for aspects of flood preparedness, impacts, financial and social support and recovery, risk factors confounding the psychological burden from the flood event such as behavioral and emotional response of the flood‐affected people are not explicitly accounted for in our study. This limitation prevents further analysis into coping strategies of the flood‐affected populations within the scope of this study. Our findings align with the evidence that flood affected‐individuals exhibit lower health‐related QoL and functioning (Fernandez et al., 2015). However, our study, based on an empirical data set is not without limitations. For instance, the survey was advertised via online media in North Rhine Westphalia due to which the response rate cannot be determined; Despite the sample size of *n* = 592, the voluntary sampling approach resulted in a response rate of 7% in Rhineland Palatinate. This response rate is comparable with the previous post‐flood surveys in Germany in the years 2002–2013 (Sairam et al., 2019). The representativeness of the survey is reported by comparing the summary statistics of the survey responses against the characteristics of the general population and by comparing the reported impacts from the respondents to official values (see, Section 3.1). Due to the absence of a control group (non‐flooded households or flooded households who did not suffer any damages) in our survey, we compared the health‐related QoL and functioning of the flood‐affected population against the general population in Germany. However it is limited by the differences in context and timing between our study and the referenced ones. Specifically, our survey was conducted during the COVID‐19 pandemic which has reduced the QoL and functioning of the general population (Klein et al., 2022; Tsai et al., 2022). Owing to the lack of a control sample, we are unable to distinguish the effects of the flood experiences from the effects of the COVID‐19 pandemic. Due to these limitations, we cannot make any concrete inferences regarding the health‐related burden due to flooding beyond the insights into the factors associated with the health‐related QoL and functioning of the flood‐affected population.

## 5 Conclusions

The study aims to derive factors associated with health‐related QoL and functioning of the flood‐affected population using an incremental linear model and three ML Models (to identify nonlinear relationships). We find that (a) socioeconomic attributes of the household such as income and ownership; (b) risk transfer options and the status of damage recovery and (c) perceived psychological burden from the flood event are associated with the health‐related QoL and functioning of the flood‐affected population. The study highlights the role of recovery and the need for strengthening the health‐related QoL of flood‐prone vulnerable populations (low socioeconomic status, poor risk transfer mechanisms). Methodological limitations of surveys in crisis research have to be taken into account. Since this was a single snapshot of the flood‐affected population, 1.5 years after the flood event, we are unable to determine the sensitivity of the indicators of health‐related QoL and functioning to time. A potential future direction is to design and implement a repeat survey targeting a longitudinal sample from the flood‐affected households to support mapping of the potential recovery pathways as illustrated by Bubeck et al. (2020).

## Conflict of Interest

The authors declare no conflicts of interest relevant to this study.

## Supporting information

Supporting Information S1

## Data Availability

The data used in this research from the 2021 flood event (excluding health‐related variables) will be made openly available after an embargo of three years as a part of the German flood damage database, HOWAS21 (Deutsches GeoForschungsZentrum GFZ, 2011). Please note that post‐flood survey data from Germany corresponding to other flood events in the past–2005, 2006, 2010, 2011, and 2013 along with instructions on how to access the data are openly available via HOWAS21. The code for ML feature extraction is openly available at https://github.com/A‐Buch/flood‐loss‐models‐4‐HCMC/tree/health‐paper.
