# Supplementary material for: Health‐Related Quality of Life and Everyday Functioning in the Flood‐Affected Population in Germany ‐ A Case Study of the 2021 Floods in West Germany
Source: Geohealth. 2025 May 29;9(6):e2024GH001135. doi: 10.1029/2024GH001135 (PMC12120565; doi:10.1029/2024GH001135)
Supplement: Supplementary file 1 — Supporting Information S1 [file GH2-9-e2024GH001135-s001.docx]

*GeoHealth*

Supporting Information for

**Health-related Quality of Life and everyday functioning in the flood-affected population in Germany - a case study of the 2021 floods in West Germany.**

Nivedita Sairam^1^, Anna Buch^2^, Marie-Luise Zenker^3^, Lisa Dillenardt^3^, Michaela Coenen^4, 5^, Annegret H. Thieken^3^ & Caroline Jung-Sievers^4, 5^

^1^ Section 4.4 Hydrology, GFZ German Research Centre for Geosciences, 14473 Potsdam, Germany

^2^ Institute of Geography, University of Heidelberg, 69117 Heidelberg, Germany

^3^ Institute of Environmental Science and Geography, University of Potsdam, Potsdam, 14476, Germany

^4^ Chair of Public Health and Health Services Research, Institute for Medical Information Processing, Biometry, and Epidemiology (IBE), Faculty of Medicine, LMU Munich, 81377 Munich, Germany

^5^ Pettenkofer School of Public Health, 81377 Munich, Germany

**Contents of this file**

Figure S1: Responses to the  WHODAS 2.0 12-item Questionnaire (interquartile range)

Figure S2: Comparison of EQ-5D VAS scores of the flood-affected population in Germany and reference value set for Germany (Interquartile range).

Figure S3: Repurchasing/repair all damaged contents after the floods (1 - all damages contents were repaired or repurchased; 6 - there are major repairs/purchases to be done)

Table S1: Questions and potential response options from the survey questionnaire

**Introduction**

Figure S1 shows a distribution of respondents based on their responses to the 12 questions in WHODAS 2.0. The 50th quantile is represented by the black circle and the line represents the interquartile range (25th and 75th percentile)

Figure S2 shows (a) gender-specific and (b) age-specific distribution of the EQ-5D VAS responses of the survey participants (in red) and value set for Germany (in green). The 50th quantile is represented by the black circle and the line represents the interquartile range (25th and 75th percentile)

Figure S3 shows a histogram of how much of the damages were replaced by the respondents at the time of the survey. The bar corresponding to 1 represents the number of respondents who replaced all of the damaged contents; 6 represents the number of respondents who still have major repairs/purchases to be done.

**
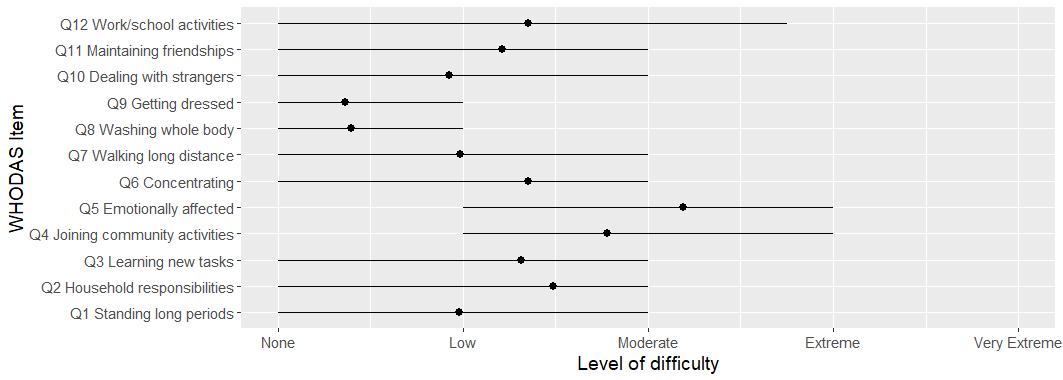
Figure S1.** Responses to the WHODAS 2.0 12-item Questionnaire (interquartile range)


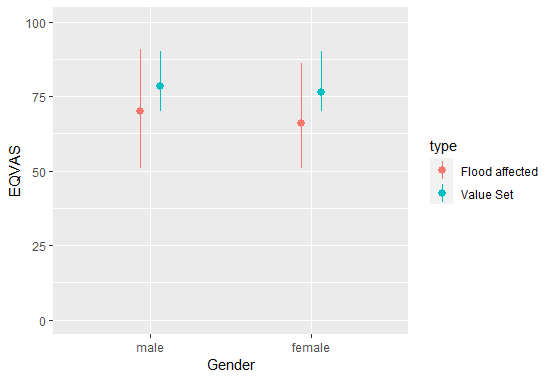

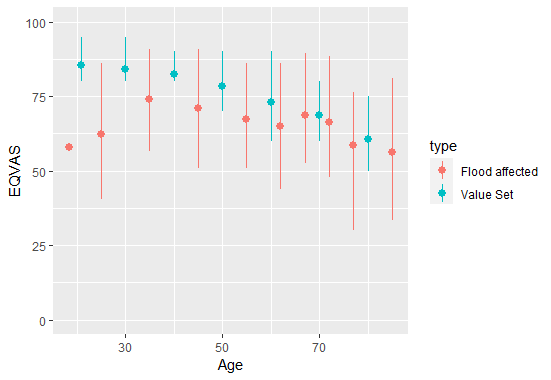


Figure S2. Comparison of EQ-5D VAS scores of the flood-affected population in Germany and reference value set for Germany (Interquartile range).


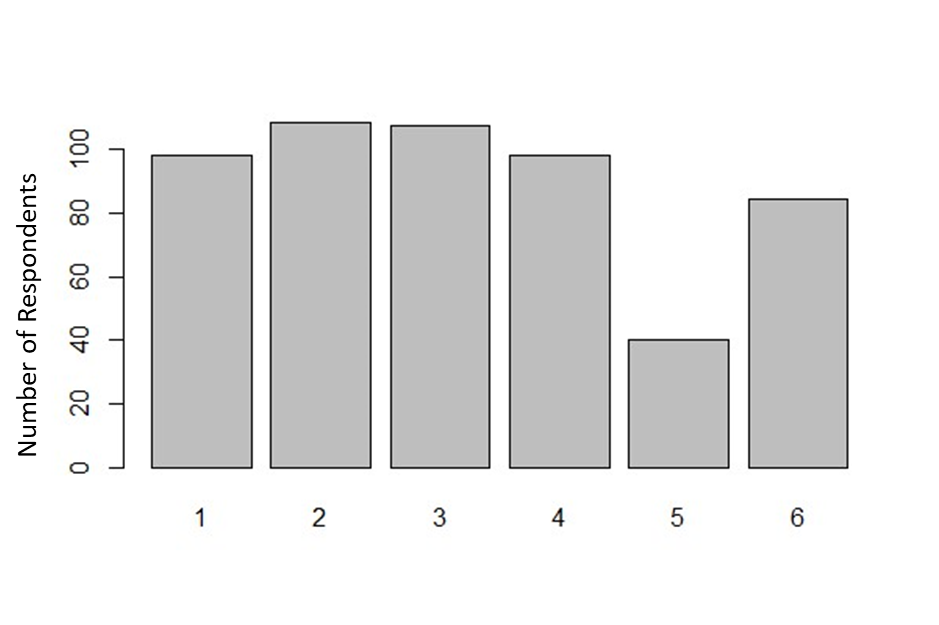


Figure S3. Repurchasing/repair all damaged contents after the floods (1 - all damages contents were repaired or repurchased; 6 - there are major repairs/purchases to be done)

Table S1. Questions and potential response options from the survey questionnaire.

| Dear participants,  Thank you for taking part in our household survey in the aftermath of the July 2021 flood event! The survey is being conducted as part of the research project funded by the Federal Ministry of Education and Research. "Scientific monitoring of the reconstruction processes after the flood disaster in North Rhine-Westphalia and Rhineland-Palatinate  - Climate Adaptation, Flood and Resilience (KAHR)". The aim of the project is to support reconstruction and rebuilding in the flooded areas. If you would like to learn more about the project, please feel free to visit our website: [hochwasser-kahr.de.](https://umfragenup.uni-potsdam.de/KAHRBefragung/hochwasser-kahr.de)  It takes about 40-50 minutes to complete the questionnaire. If you do not want to complete a question, please continue with the questionnaire anyway. You can then use the option "No answer" instead.  The questionnaire contains concrete questions about the 2021 flood disaster. This can create a sense of reliving. For the severely affected, this may be very demanding. We understand this and would like to ask you to participate in the survey only if you feel sufficiently resilient. If you feel the need to talk to someone about what you have experienced during the survey or in general, you can contact the BDP (Professional Association of German Psychologists) - Flood Hotline: ☎ 0800 7772244. You can find an overview of regional offers here: [www.psychiatrie.de/flutkatastrophe-in-deutschland-seelische-unterstuetzung-fuer-betroffene-angehoerige-und-helfende.](http://www.psychiatrie.de/%EF%AC%82utkatastrophe-in-deutschland-seelische-unterstuetzung-fuer-betro%EF%AC%80ene-angehoerige-und-helfende) A hotline has been set up for those affected by the 2021 flood disaster in the Ahr valley. You can reach it from Mon-Fri between 8 a.m. and 5 p.m. at 0800- 7295729. A collection of offers of help find those affected and [helpers at the following link: https://www.flutkatastrophe-rlp.de/hilfe-finden](https://www.flutkatastrophe-rlp.de/hilfe-finden) | |
| --- | --- |
| **Variable** | **Question** |
| gender | Which gender do you feel you belong to?  Male;  Female;  Diverse;  not specified |
| age | How old are you?  18:19;  20:29;  30:39;  40:49;  50:59;  60:64;  65:69;  70:74;  75:79;  >= 80 |
| household_size | How many people, including you, live in your household? |
| income | What is the approximate total monthly net income of your household in EURO?  *This refers to the sum of all income that remains AFTER deduction of taxes and social security contributions. For farmers, self-employed and freelancers, what is the average monthly net income of your household minus your operating costs?* Under 900€;  900€ : 1299 €;  1300€ : 1499€;  1500€ : 1999 €;  2000€ : 2599€;  2600€ : 3599€;  3600€ : 499 €;   >= 5000€;  not specified |
| socioeconomic | Computed based on ownership, footprint size, building type, education (Plapp et al. 2003) |
| education | What is your highest level of education?  No school-leaving qualification;  Hauptschulabschluss or Volksschule;  Realschulabschluss/Mittlere Reife;  Completed vocational training;  Fachhochschulreife/Fachabitur Hochschulreife/Abitur;  Meister/ Techner;  University degree;  Doctorate;  Other ________________;  Not specified |
| ownership | Are you renting or do you own the house or flat?  *Part owners are considered owners (e.g. joint ownership of married couples, owners associations, etc)*  I live in a rented house;  I am the owner of the flat (condominium);  I am the owner of the house; |
| building_type | Which description best fits your home?  Detached house  Semi-detached house  Terraced house  Farm house (residential building adjoins a barn, threshing floor or stable: however the farmhouse need no longer be used for agricultural purposes)  Detached or semi-detached house built into the slope (terrace house)  Detached apartment building (up to 6 storeys)  High-rise building (detached with more than 6 storeys)  Apartment block (i.e large blocks with several house entrance/house numbers) |
| building_quality | Overall, how would you rate the quality of construction and fit-out of the building BEFORE the July 2021 event? *This refers to the quality of the building fabric, plastering, doors, windows etc.*  1 - very good, high quality  2  3  4  5  6 - Very poor, in need of renovation  Not specified |
| building_footprint | Please enter the approximate size of your apartment/house. *Please enter the area in SQUARE METRES in the input field. Note: The square metre number is also stated in the rental contract*. |
| flood_experience | How often have you personally been affected by heavy rain or flooding before the event in July 2021? *That is, how often has your home been affected by flooding? If you have moved in the past, this may also have been at a previous place of residence.*  Once;  Twice;  Three times;  Four times;  More than four times; |
| warning_source | Please think back to the hours before the event. How did you become aware that the danger of flooding will become severe for you? *A storm warning refers to warnings of thunderstorms and heavy rain. A flood warning refers to flooding and water levels at water level gauging stations. (Multiple answers possible)*  Severe weather or flood warning by local authorities or civil protection (e.g. fire brigade or police)  Warning by evacuation call  Radio  Television (e.g. weather report or teletext)  Daily newspaper  Weather app  Severe weather app (e.g. Katwarn, NINA, warning weather app)  Siren or loudspeaker truck  Self-research on the internet  Social networks on the internet (e.g. Facebook, Twitter)  Through others, e.g. neighbours, acquaintances, colleagues, friends etc. (e.g. personal conversation, phone call, e-mail) Through my employer  Through care or educational institutions (e.g. school, day care centre) |
| **Questions about what you have experienced and how you are affected can trigger feelings of re-experiencing. We would therefore like to draw your attention once again to individual counselling and support services:**  If you feel the need to talk about what you have experienced, you can contact the BDP (Berufsverband Deutscher Psychologinnen und Psychologen - Professional Association of German Psychologists).  **- Flood hotline:** ☎ 0800 7772244. You can find an overview of regional services here: [www.psychiatrie.de/flutkatastrophe-in-](http://www.psychiatrie.de/%EF%AC%82utkatastrophe-in-)deutschland-seelische-unterstuetzung-fuer-betroffene-angehoerige-und-helfende. A hotline has been set up for those affected by the 2021 flood disaster in the Ahr valley. You can reach it from Mon-Fri between 8 a.m. and 5 p.m. at 0800-7295729. A collection of offers of help can be found at the following link: <https://www.flutkatastrophe-rlp.de/hilfe-finden> | |
| warning_time | How many hours before the flooding did the warning reach you or you became aware of the danger yourself? *If there were several warnings, the question refers to the first warning that reached you. Please enter the number of hours in the input field.*  Number of hours between the warning and the occurrence of the event: |
| measures | What mitigation measures did you take just before and during the 2021 flood to protect your house/apartment? *Please select the measures you have implemented.*  Just before or during the 2021 flood, did you...  Secure documents and valuables  Raise or move furniture and movable objects to a safe place  Secure oil tanks or containers with other hazardous substances  Pump water out or skimmed off  Bring animals to safety  Drive vehicles onto flood-proof terrain  Protect the building itself against water penetration, e.g. by sealing doors, windows, drains and other openings.  Divert water through measures on the site, e.g. digging trenches, building sandbag walls, mobile walls, etc.  Get help from outside (fire brigade, friends, etc.)  Unplug and secure electrical devices, tape off sockets  Secure or dismantle electrical installations, permanently installed building parts (doors etc.) switch off Gas/electricity  Or was gas/electricity cut off centrally by municipal utilities?  I have not implemented any of these measures |
| insurance | Did you take out an insurance against flooding before the 2021 event? Yes; No; Not specified |
| water_depth | Approximately how high was the water in the highest affected floor, measured from the floor in centimetres?  On which floors including the cellar was the water in your house when the water level was the highest? *Please do not include outbuildings and detached garages. The attic also counts as a floor (Multiple answers possible)*  Basement  Ground floor  1 Floor  2 Floor  3 Floor  4 Floor  Top Floor |
| duration | How many hours in total did the water stand in the house?  *This refers to the time until the water was drained, pumped out and out of the house again. An approximate estimate is sufficient.*  *1 day = 24 hours, 2 days = 48 hours, 3 days = 72 hours, 4 days = 96 hours, 5 days = 120 hours, 6 days = 144 hours,*  *7 days = 168 hours, 8 days = 192 hours, 9 days = 216 and 10 days = 240 hours*  Indication in hours:  Longer than 10 days. |
| contamination | Was your affected property additionally contaminated by the following contaminants?  *(Multiple answers possible)*  Chemicals, paints, varnishes, pesticides, small quantities of engine oil  Waste water or faeces  Heating oil  Petrol  No, no additional pollution from this Stoffe. No indication |
| velocity | How strong was the water flow in the immediate vicinity of your house?  *Please select a number between 1 (Calm flowing) and 6 (Wild/Torrential).*  1- 6;  No flow rate No indication |
| human_stability | What do you think:  would an average man be able to stand upright effortlessly in this flood in the immediate vicinity of your house, would he have had to strain to remain standing, or would he have been swept away?  *The most dangerous point in time means the highest flow velocity/current speed.*  A man could have stopped effortlessly.  A man would have had to strain to remain standing. A man would have been swept away.  The water was too deep to stand.  Not specified |
| evacuation_b | Did you have to leave your flat or house due to the flood?  *It is considered "yes" if at least one household member had to leave the house or flat.*  Yes, I had to leave the house BEFORE or DURING the flood.  Yes, I had to leave the house AFTER the floods.  No, I did not have to leave the house.  Not specified |
| evacuation_a |  |
| building_loss | If you add up the costs (material and labour) for all the necessary repair work on and in the building: What was the total amount of damage done to your building?  *This also includes costs for rental equipment such as building dryers, dehumidifiers, etc.*  Amount of total damage to the building in EURO |
| content_loss | How high do you estimate the total costs necessary to restore your damaged household goods?  Please state the total amount for all damaged objects in EURO. |
| injury_self | Have you suffered a serious injury yourself or become seriously ill? Yes; No |
| injury_family | Have any family members or close friends of yours suffered a serious injury or become seriously ill? Yes; No |
| worried_safety | Were you at any time during or after the flood uncertain about the safety or whereabouts of family members or close friends? Yes; No |
| death_family | Have you lost a family member or close friend due to a death as a result of the floods? Yes; No |
| repurchase_contents | Please compare your household contents BEFORE the overflood and their current condition (NOW). Have you replaced the damaged household goods in the meantime or do the household goods still have glaring defects and gaps as a result of the flood?  1 - Completely re-procured 2  3  4  5  6 - Significant gaps and deficiencies  Not specified |
| repair_building | Please compare the building BEFORE the flood and its current condition (NOW)  1 - Completely restored 2  3  4  5  6 - Still considerable damage  Not specified |
| freq_thoughts | How often have you thought about the damage event of July 2021 in the last 6 months?  *This refers to the period since approx. April 2022.*  Several times a day Daily  Several times a week or almost daily  About once a week or several times a month About once a month  A total of one to four times in the last six months Not once in the last six months  Not specified |
| psychological_burden | How much does the flood event of July 2021 still bother you today?  1 - It doesn't bother me at all any more/I feel the same as before the event.  2  3  4  5  6 - It still weighs heavily on me.  Not specified |
| claims_completion | Has the loss compensation already been completed?  *This means whether the formalities and payments have been completed or whether the process is still ongoing.* Yes; No  Not specified |
| claims_satisfaction | How satisfied were you overall with the claims settlement?  1 - Very satisfied  2  3  4  5  6 - Very dissatisfied  Not specified |
| immediate_aid | In what form have you received financial compensation?  *(Multiple answers possible)*  Immediate help  Oil spill response assistance  Hardship reconstruction assistance  Private donations  Insurance benefits  Rent reduction;  No financial compensation received  No indication |
| oilspill_response |  |
| hardship_assistance |  |
| private_donations |  |
| insurance_benefit |  |
| rent_reduction |  |
| social_friends | Have you also received help in the form of e.g. donations in kind and/or support staff from the following parties?  *Please name the THREE most important.*  Help from family and relatives  Help from neighbours and friends  Help from volunteers  Help from the fire brigade, THW or police  Help from rescue or welfare organisations, e.g. the German Red Cross, Arbeiter-Samariterbund, etc.  Help from church institutions  Help from the community  Tax relief Other, namely: |
| social_organization |  |
| social_community |  |
| EQ-5D VAS | Please assess how good or bad your health is TODAY.  *Please use the slider for this.*  0-------------------------100  Please rank your health between the two numbers 100 = The best health you can imagine and 0 = The worst health you can imagine. |
| WHODAS 2.0 | How many difficulties did you have in doing the following activities during the last month, i.e. the last 4 weeks, because of your health problems?  The options are: None, Low, Moderate, Strong, Very Strong/Not possible,  Not specified  Did you have any difficulties ...  ... to stand for a longer time (approx. 30 minutes)?  ... To meet your budgetary obligations?  ... learn new tasks (e.g. learn to get to a new place you didn't know)?  ... participate in social activities (such as festivities, religious or other activities) in the same way as everyone else?  How much emotional strain has your health condition put on you?  ... to concentrate on something for 10 minutes?  ... walk a longer distance (approx. one kilometre)?  ... wash your entire body? |
| **Thank you for your participation!**  We thank you very much for taking the time to complete this survey. We wish you personally, as well as the entire region, much strength for the reconstruction. If you have any questions, please contact: [xxxxxxx@xy.de](mailto:xxxxxxx@xy.de). Questioning can help to process the experience, but can also lead to the event becoming a burden again. Please seek help in this case. If you need psychological acute help, please contact the BDP (Professional Association of German Psychologists) - Flood Hotline: ☎ 0800 7772244. Here you can find an overview of regional offers: [www.psychiatrie.de/flutkatastrophe-in-deutschland-seelische-unterstuetzung-fuer-betroffene-angehoerige-und-](http://www.psychiatrie.de/%EF%AC%82utkatastrophe-in-deutschland-seelische-unterstuetzung-fuer-betro%EF%AC%80ene-angehoerige-und-)hellping. A hotline has been set up for those affected by the 2021 flood disaster in the Ahr valley. You can reach it from Mon-Fri between 8 a.m. and 5 p.m. at 0800-7295729. Your data has now been saved. You can now close the window. | |
